# Ruptured Sinus of Valsalva Aneurysm With Fistulous Communication to the Pulmonary Artery Revealing Behçet’s Disease

**DOI:** 10.7759/cureus.97945

**Published:** 2025-11-27

**Authors:** Anpu M Devassy, Joanne Shannon

**Affiliations:** 1 Renal Medicine, Frimely Park Hospital NHS Foundation Trust, Surrey, GBR; 2 Cardiology, Frimely Park Hospital NHS Foundation Trust, Surrey, GBR

**Keywords:** aortic valve replacement (avr), behçet’s disease, fistula aortic sinus to pulmonary artery, immunosupression, ruptured sinus of valsalva, sinus of valsalva aneurysm

## Abstract

We present the case of a 32-year-old South Asian male patient with a four-day history of cough, fever, and haemoptysis who was found to have a ruptured sinus of Valsalva aneurysm (SoVa) with a fistulous connection to the pulmonary artery. Examination revealed tachycardia and a loud murmur with thrills over the pulmonary area, along with features of pulmonary oedema. Nasopharyngeal polymerase chain reaction (PCR) test confirmed COVID-19 infection. Transthoracic echocardiography demonstrated turbulent left-to-right shunting at the great artery level, mild functional mitral and tricuspid regurgitation, and elevated pulmonary artery systolic pressure. Computed tomography pulmonary angiography identified a ruptured SoVa with a fistulous connection to the pulmonary artery, associated right upper lobe pulmonary embolism, and pulmonary congestion.

Initial management included corticosteroids and antivirals for COVID-19, broad-spectrum antibiotics for suspected infective endocarditis, and diuretics. Progressive cardiac dysfunction necessitated percutaneous closure of the fistula using an 8-mm occluder device, achieving prompt haemodynamic stabilisation. Subsequently, the patient developed severe aortic regurgitation, aortic root dilatation, and right ventricular dysfunction, requiring urgent aortic root replacement with a tissue prosthesis and right ventricular repair.

Persistent postoperative pyrexia and elevated inflammatory markers despite sterile blood cultures prompted histopathological evaluation, which revealed necrotising granulomatous inflammation with multinucleated giant cells, which couldn't rule out giant-cell aortitis. A rheumatological review uncovered recurrent oral and genital ulceration, confirming Behçet’s disease as the underlying aetiology.

Immunosuppressive therapy with corticosteroids, infliximab, and azathioprine, together with treatment for latent tuberculosis (TB), resulted in full clinical remission. At one-year follow-up, ventricular function and prosthetic valve performance remained preserved with no evidence of recurrent aneurysmal change or inflammatory activity.

This case highlights the complex interplay between structural cardiac pathology, infection, and systemic vasculitis, underscoring the importance of multidisciplinary evaluation and targeted immunosuppressive therapy.

## Introduction

A ruptured sinus of Valsalva aneurysm (SoVA) is a rare congenital or acquired cardiac anomaly that can precipitate acute heart failure, cardiogenic shock, or even sudden death, making prompt recognition and management essential. We present a diagnostically challenging case of a young male who initially presented with a ruptured SoVA in the context of COVID-19 pneumonitis and pulmonary oedema, which precluded immediate surgical intervention. He underwent percutaneous closure as a stabilising measure before ultimately requiring aortic root replacement and right ventricular outflow tract (RVOT) repair.

Despite technically successful interventions, the patient developed prolonged postoperative fever, persistent inflammation, and new valvular pathology without identifiable infection. Extensive microbiological, autoimmune, and histopathological testing were inconclusive. The later development of mucocutaneous ulcerations and a dramatic response to corticosteroid therapy led to a final working diagnosis of Behçet’s disease.

This case illustrates the diagnostic overlap between infection and vasculitis in structural cardiac disease and highlights the importance of considering vasculitis in complex postoperative or inflammatory presentations. Early recognition and targeted immunosuppressive therapy are critical to preventing catastrophic vascular complications and improving survival in vascular Behçet’s disease.

## Case presentation

**Figure 1:**
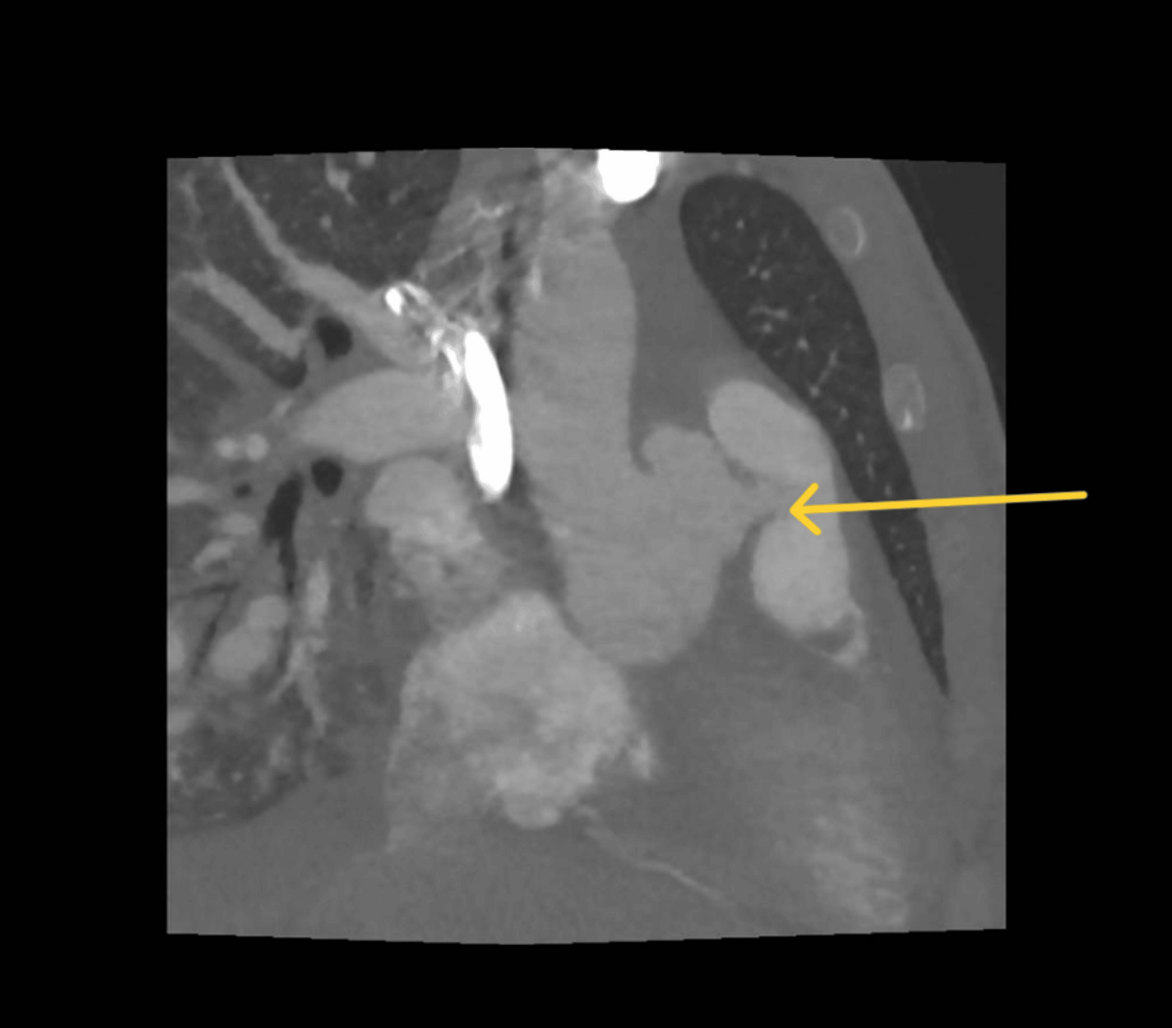
Sagittal CT scan of the lungs demonstrating a sinus of Valsalva aneurysm with a fistulous connection to the pulmonary trunk.

**Figure 2:**
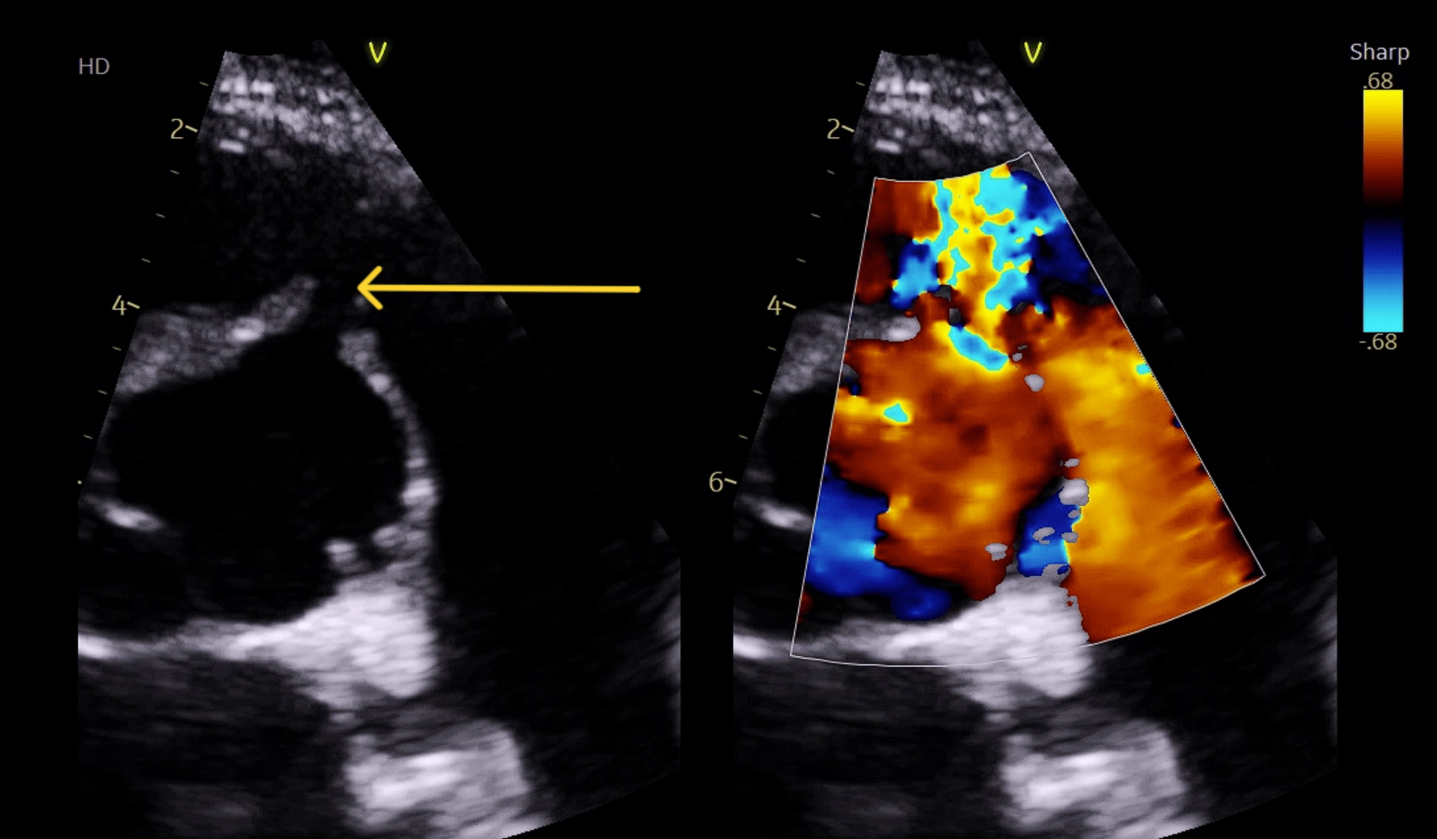
Transthoracic echocardiography demonstrating abnormal turbulent left-to-right color flow at the level of the aortic root.

A 32-year-old South Asian male patient with no significant past medical history presented with acute dyspnoea and chest discomfort. Initial transthoracic echocardiography and CT imaging revealed a ruptured SoVA with a left-to-right shunt into the RVOT (Figures 1, 2, and Video 1). Concurrent findings included pulmonary oedema and radiological features consistent with COVID-19 pneumonitis. Given the high perioperative risk related to underlying pulmonary pathology, the case was discussed at the adult congenital heart disease multidisciplinary team (ACHD MDT), and the decision was made to proceed with percutaneous closure as a temporising measure. An 8 mm muscular ventricular septal defect (VSD) occluder was successfully deployed, with the cardiothoracic surgical team on standby. 

**Video 1:** Echocardiogram demonstrating a fistulous connection between the aortic root/sinus of Valsalva and the pulmonary trunk/artery.

During early recovery, the patient developed pleuritic chest pain with saddle-shaped ST elevation and low-grade fever. A diagnosis of pericarditis was made. Recurrent febrile episodes led to further septic screening, which remained negative. A transoesophageal echocardiogram showed severe aortic regurgitation without vegetations. Due to clinical instability and echocardiographic progression, including torrential aortic regurgitation, increasing aortic root diameter, RVOT obstruction, and elevated right ventricular systolic pressure, urgent surgical intervention was undertaken. The patient underwent aortic root replacement (Bentall procedure), occluder device explantation, and RVOT patch reconstruction for a perforation identified intraoperatively. Key investigations and findings are provided in Table 1 below.

**Table 1:** Key investigation results

Investigation / Procedure	Key Findings / Outcome
Percutaneous closure of ruptured sinus of Valsalva aneurysm (SoVA) using an 8 mm Abbott muscular VSD occluder (Abbott Laboratories, Abbot Park, IL, US) under general anaesthesia	A 7-mm ruptured SoVA with fistulous communication between the aorta and pulmonary trunk was successfully closed. No residual shunt post deployment. Haemostasis achieved with ProStyle closure (Abbott Laboratories).
Intraprocedural transoesophageal echocardiogram (TOE)	Normal biventricular systolic function; trileaflet aortic valve with mild regurgitation; large SoVA (~3 cm) forming a short aorto-pulmonary fistula; no dissection or vegetation. Post procedure: well-seated device with preserved pulmonary valve function and unobstructed right ventricular outflow tract (RVOT).
Transthoracic echocardiography (serial)	Post device: progressive aortic root dilatation (up to 49 mm) with severe aortic regurgitation; mild-to-moderate RVOT obstruction due to device protrusion; preserved left ventricular (LV) systolic function. Following Bentall surgery: satisfactory prosthetic aortic valve function, mild mitral regurgitation (MR), moderate–severe tricuspid regurgitation (TR), no significant pulmonary regurgitation (PR), and mildly impaired right ventricular (RV) function.
Cardiac MRI	Successful occlusion of ruptured SoVA with no residual shunt (Qp:Qs ≈ 1.1). Occluder protruding into RVOT, causing mild flow acceleration (~2 m/s). Functionally bicuspid aortic valve with moderate eccentric regurgitation (regurgitant fraction (RF) 22%). Mild pericardial inflammation and small effusion; normal biventricular size and function.
Aortic root replacement (Tissue Bentall) with occluder removal and RVOT reconstruction	Severely disrupted aortic root with a large 4 × 4 cm fistulous perforation into RVOT containing an occluder. Aortic root replaced using a 23 mm Perimount Magna Ease valve (Edwards Lifesciences, Irvine, CA) within a 26 mm tube graft; RVOT repaired with bovine pericardial patches. Post-cardiopulmonary bypass (CPB) TOE: trivial aortic regurgitation (AR), moderate MR, moderate–severe TR, preserved LV function, patent coronaries, and no significant PR.
Post-operative echocardiography	Bioprosthetic aortic valve replacement (AVR) was well seated with normal haemodynamics; mild–moderate MR; moderate TR; preserved LV systolic function; mild RV dysfunction; no RVOT obstruction.
CT/PET-CT imaging	Fluorodeoxyglucose (FDG) uptake around the sternotomy and ascending aorta was consistent with postoperative inflammation rather than infection. Stable perigraft collection with minimal gas; no abscess or mediastinitis. No pulmonary embolism or infarct.
Follow-up echocardiography (late)	Multiple small mobile masses attached to pulmonary valve leaflets and RVOT wall (5–6 mm), representing vegetations or thrombus; mild pulmonary stenosis and regurgitation; preserved biventricular systolic function; well-functioning bioprosthetic aortic valve without regurgitation.

Postoperative recovery was complicated by persistent fever and inflammatory markers without microbiological confirmation of infection. Imaging identified a new lesion on the pulmonary valve concerning for vegetation or thrombus. Antimicrobial therapy was broadened empirically, and anticoagulation with low molecular weight heparin was initiated. A positive interferon-gamma release assay raised suspicion for latent tuberculosis (TB), and empirical anti-TB therapy was commenced. Antifungal treatment was also initiated in light of clinical deterioration and ongoing pyrexia. A concise investigation list of immunology/serology/ microbiology profile performed on the patient is provided in Table 2 below.

**Table 2:** Immunology, serology, and microbiology profile of the patient

Category	Tests Performed	Key Results	Interpretation
Immunoglobulin profile	IgA, IgG, IgM, IgG subclasses	Normal total IgG, IgA, IgM; low IgG2 subclass	Mild isolated IgG2 deficiency
Autoantibodies	dsDNA, MPO, PR3, ENA (RNP, Jo-1, Scl-70, centromere, SSA, SSB), RF	All negative	No evidence of systemic lupus erythematosus, ANCA-vasculitis, or connective-tissue disease
Antiphospholipid antibodies	β2-glycoprotein I (IgG, IgM), anticardiolipin (IgG, IgM)	All negative	No antiphospholipid syndrome
Lymphocyte subsets	CD3, CD4, CD8, CD19, NK cells	Mild B-cell lymphopenia and slightly reduced CD4%; otherwise preserved T-cell and NK profiles	Minor immune dysregulation; cellular immunity largely intact
Mast-cell/allergy marker	Tryptase	Within reference range	No evidence of mast-cell activation
Infectious screen	HIV, hepatitis B/C, syphilis, *Legionella*, cytomegalovirus, Epstein–Barr virus, IgM, *Brucella*, *Coxiella*, Methicillin-Resistant *Staphylococcus aureus*, carbapenemase-producing organism, antistreptolysin O, malaria	All negative	No active infection identified
Mycobacterial/fungal studies	T-Spot tuberculosis (TB), BDG assay, 16S/18S polymerase chain reaction (PCR)	Positive T-Spot TB, positive β-D-glucan, but all tissue PCR and stains were negative.	Suggests immune cross-reactivity or latent infection rather than active mycobacterial/fungal disease.
Microbiology	Multiple blood cultures, aortic tissue cultures	All negative	No microbiological confirmation of infective endocarditis
Clinical correlation	Fever and vegetations were unresponsive to antibiotics, but rapid improvement on corticosteroids.	Overall pattern favours sterile immune-mediated endocarditis/aortitis over bacterial infection.	

Histopathology of the aortic root showed necrosis with inflammatory infiltrates but no definitive evidence of infection, plasma cell infiltration, or vasculitis (Table 3). IgG4 staining was negative. Rheumatology input was sought due to a history of recurrent oral and genital ulceration. In the context of systemic inflammation and cardiovascular involvement, a working diagnosis of Behçet’s disease was made. The patient responded promptly to intravenous corticosteroids, but fevers recurred on transition to oral therapy. Immunosuppression was escalated with infliximab and azathioprine following TPMT testing, resulting in rapid resolution of symptoms and inflammatory markers.

**Table 3:** Histopathology and tissue findings

Investigation/Source	Key Findings	Interpretation/Conclusion
Aortic valve and wall (operative specimens)	Necrotic, partially calcified valve tissue with mixed inflammatory infiltrate (neutrophils, eosinophils, histiocytes, fibroblasts). Aortic wall showing focal intimal inflammation with smooth-muscle loss in the media; no laminar necrosis or classical giant cells.	Features of active endocarditis involving the aortic valve and active aortitis with medial destruction. Giant cells likely reactive to necrosis; a primary giant-cell aortitis cannot be excluded.
Special stains and molecular studies	Gram, periodic acid–Schiff, Grocott, Wade–Fite, Warthin–Starry, spirochaete, and Bacillus Calmette-Guérin (BCG) immunostains were all negative. 16S and 18S rRNA polymerase chain reaction (PCR), fungal and bacterial panels were negative.	No histological or molecular evidence of infective organisms.
External histopathology review (ID reference centre)	Ulceration and necrosis of the aortic valve with neutrophilic and histiocytic inflammation. Aortic tissue with intimal fibrosis, medial necrosis, mixed inflammatory infiltrate, and scattered mononuclear cells. No granulomas or vasculitis.	Findings consistent with active non-specific aortitis/endocarditis. Likely immune-mediated inflammation; infection not completely excluded despite negative microbiology.
Multidisciplinary correlation	Valve and aortic changes occurred in a background of systemic inflammation with negative cultures and a dramatic steroid response.	Overall impression: immune-mediated aortitis/endocarditis (possible Behçet-spectrum vasculitis).

Given the risk of TB reactivation, isoniazid monotherapy was initiated in place of rifampicin to avoid drug interactions with anticoagulation. Anticoagulation was transitioned to apixaban with plans for community monitoring. Additional findings during admission included vertebral osteopenia on dual-energy X-ray absorptiometry scan secondary to corticosteroid use, borderline thyroid function abnormalities, and transfusion-requiring anaemia. The patient was discharged on infliximab, azathioprine, isoniazid, and apixaban, with outpatient follow-up arranged with rheumatology, infectious diseases, and cardiology teams.

At one-year follow-up, the patient remained clinically stable, afebrile, and functionally active with normal cardiac function and well-controlled inflammatory disease activity.

## Discussion

SoVA represents an uncommon but life-threatening cardiovascular event. While congenital causes predominate, acquired aetiologies such as infection, trauma, or inflammatory vasculitis should also be considered, particularly when the presentation is atypical or culture-negative. In this case, a young male developed a ruptured SoVA in the context of COVID-19 pneumonitis and pulmonary oedema. Initial stabilisation was achieved through percutaneous closure, followed by definitive aortic root replacement and RVOT reconstruction once haemodynamic stability was restored.

Persistent postoperative inflammation, prolonged fever, and negative cultures created diagnostic uncertainty. The later appearance of mucocutaneous ulceration and a dramatic response to corticosteroids and biologic therapy established Behçet’s disease as the underlying aetiology. Behçet’s disease is a multisystem vasculitis that can affect both arteries and veins of any size, leading to thrombosis, stenosis, or aneurysm formation [1,2]. Cardiovascular involvement occurs in less than 5% of patients but carries significant morbidity and mortality, with complications including endocarditis-like lesions, intracardiac thrombus, valvular insufficiency, and aortic or SoV aneurysm rupture [3-5].

The pathogenesis involves neutrophilic infiltration and destruction of the vessel media, resulting in wall weakening, aneurysm formation, and potential rupture [6]. Arterial involvement in Behçet’s disease may affect the aorta, coronary, or pulmonary arteries, with aneurysmal complications reported in multiple vascular territories [7-9]. Pulmonary artery aneurysms, though rare, are a recognised manifestation of vascular Behçet’s disease and may respond to anti-TNF therapy [9]. Similarly, the presence of aortic or coronary aneurysms reflects widespread vasculitic activity rather than isolated mechanical failure [10].

Surgical or endovascular repair in vascular Behçet’s disease carries a high risk of postoperative pseudoaneurysm formation or graft detachment, particularly when performed without immunosuppressive therapy [11]. Therefore, perioperative corticosteroids and cytotoxic or biologic agents are essential to control inflammation and reduce recurrence. Our patient’s favourable response to corticosteroids, azathioprine, and TNF-α inhibition aligns with current evidence supporting early immunosuppression in severe or refractory vascular Behçet’s disease [4,8,11].

From a diagnostic standpoint, distinguishing vasculitic inflammation from infective or sterile postoperative inflammation is challenging. Key features suggesting vasculitis include persistent systemic inflammation with negative microbiology, multi-site vascular involvement, and mucocutaneous lesions. In such cases, close collaboration between cardiology, cardiothoracic surgery, rheumatology, and infectious disease specialists is vital to ensure accurate diagnosis and optimal outcomes.

## Conclusions

This case highlights the diagnostic and therapeutic complexity of managing a ruptured SoVa complicated by systemic inflammation secondary to Behçet’s disease. The overlap between infection, postoperative inflammation, and vasculitis required close multidisciplinary collaboration across cardiology, rheumatology, infectious diseases, and cardiothoracic surgery to achieve diagnostic clarity and optimal management.

Behçet’s disease, though uncommon, should be considered in patients with sterile cardiovascular inflammation, recurrent fever, or unexplained aneurysmal changes, particularly when associated with mucocutaneous ulceration. This case reinforces the need to consider vasculitis early in postoperative inflammation unresponsive to antibiotics, especially in patients of high-risk ethnic backgrounds or with mucocutaneous symptoms. Early recognition and timely immunosuppressive therapy are critical to preventing life-threatening vascular complications. This case also underscores the value of a stepwise treatment strategy, using percutaneous closure as a bridge to definitive surgery, and highlights the importance of perioperative immunosuppression in minimising postoperative inflammatory complications in vasculitic conditions.
